# Supplementary material for: Over-indebtedness and health in Switzerland: A cross-sectional study comparing over-indebted individuals and the general population
Source: PLoS One. 2022 Oct 11;17(10):e0275441. doi: 10.1371/journal.pone.0275441 (PMC9553041; doi:10.1371/journal.pone.0275441)
Supplement: Attachment — Submitted filename: renamed_509f2.docx [file pone.0275441.s003.docx]

Zurich, 28 March 2022

Dear Dr. Useche,

We have now revised our manuscript entitled

**Over-indebtedness and health in Switzerland: a cross-sectional study comparing over-indebted individuals and the general population**

**(PONE-D-21-26584)**

Please find attached the revised manuscript along with our replies to the comments and points raised by the reviewers. All corrections and additions are highlighted in yellow. The deleted words, sentences or paragraphs are not marked.

**Reviewer’ comments and authors’ replies**

**Reviewer #1:**

“Please clarify the recruitment process and response rate in both surveys. Did both surveys include only respondents from the Canton of Zurich? If not, to what extent are the four official debt advisory centers in the Canton of Zurich and clients presenting to these centers representative for centers and clients across Switzerland? Please clarify the study population early in the methods section. What eligibility criteria was applied in the Swiss Health Survey? What were sources and methods of selection of participants in the Swiss Health Survey? How was the subsample of adult respondents of the Swiss Health Survey selected? In Table 1 the authors mention that the Swiss Health Survey data was weighted. How was the weighting process carried out?”

*Clarifications on the data collection among over-indebted individuals have been made now (see yellow marked comments in the Methods section). In fact, we have completely reformulated and shortened the first part of the Methods section (“Data and study population”).*

*For further information about the nationally representative and publicly available data of the Swiss Health Survey and the sampling and weighting procedures see the following webpage and/or publication:*

- [*https://www.bfs.admin.ch/bfs/de/home/statistiken/gesundheit/erhebungen/sgb.html*](https://www.bfs.admin.ch/bfs/de/home/statistiken/gesundheit/erhebungen/sgb.html)
- *Federal Statistical Office FSO (2018). Die Schweizerische Gesundheitsbefragung 2017 in Kürze. Konzept, Methode, Durchführung. Bundesamt für Statistik BFS, Bern.*

**Background**

“Please clarify the objectives of the study. What are the key objectives of the study? Was the study conducted to compare sociodemographic characteristics of those over-indebted to the general population in the Canton of Zurich (166) and/or to study the prevalence of health problems and association between over-indebtedness and health (146-153)?”

*In summarizing and clarifying the research interest of the present study three research questions to be addressed have now been explicitly formulated and inserted at the end of the Background section.*

**Methods**

“In the methods section, the authors state that descriptive statistics, bivariate and multivariate association analyses were performed (266-274). From my point of view, the authors should revise this section to clarify what measures they used to achieve which purpose. The authors state “multiple adjusted odds ratios (aOR) as measures of the relative risk” (273) which, in my view, is not a correct description of the statistical measure. What significance level was applied? How were missing data addressed? The different numbers of cases per model (Table 3, Table 4) indicate that individual cases were excluded from the multiple logistic regression analyses. Did the authors conduct any sensitivity analyses?”

*We have reformulated almost the entire subsection “Analyses” in the Methods section accordingly. As regards some specific points raised by the reviewer we’d like to comment as follows:*

- *Odds ratios are commonly used and reported measures of association between exposures and outcomes and at least in case of rather uncommon or rare diseases or outcomes good proxy measures of the relative risk. However, the relative risk or risk ratio (RR) compares the risk of an event or disease in an exposed and a non-exposed group of individuals while the odds ratio (OR) compares the probability of an event or disease with the probability of the non-occurance of the event or disease. When an outcome occurs in less than 10% of the unexposed population the OR provides a reasonable approximation of the RR, but when an outcome is more common the OR overestimates the RR. Since OR and RR in fact are not exactly the same measures and since we calculated logistic regression analyses which do not provide RRs we have decided to forego comparing odds ratios with measures of relative risk.*
- *The significance level as usual was 5% (p<=.05), indicated in the Tables 3 and 4 as 95% confidence invervals. However, we have additionally provided higher significance levels of 1% and 1‰ (**p<.01, ***p<.001).*
- *There were only very few missing cases (less than 1%) except for one association analysis and health outcome (depression; less than 3%). Missing cases therefore were not addressed at all. Excluding these cases from the association analyses did not result in any significant loss of robustness or generalizability of the findings.*
- *Sensitivity analyses were not performed since logistic regression analyses were performed and not linear regression analyses using standardized regression coefficients. However, group-specific prevalence rates were additionally provided and indicated in Tables 3 and 4 in order to better or more adequately evaluate the health risks (odds ratios) of the exposed (over-indebted) individuals, and to assess the influence of the covariates considered.*

“In order to clarify the methodological approach, I suggest to present key elements of the study design early in the paper, and to provide detailed information later. From my perspective, the authors could focus more on describing key characteristics of the methodological approach (study design, setting, participants, variables, study size, variables, statistical methods) and reduce additional details (e.g. 207-214) to avoid redundancy (“As already mentioned before…” 215) and, above all, make it easier for readers to understand which methods were used. The authors, for instance, list measures of over-indebtedness (178ff.) in the methods section and describe in detail why primary data was collected (172-194). Instead, the authors could briefly summarize these aspects and elaborate on relevant aspects in the discussion section, e.g. with regards to potential limitations of the study design or measures used in the study.”

*We fully agree with the reviewer’s suggestion and have strongly shortened, realigned and focused the Methods section (or rather the ‘Data and study population’ subsection). More specifically, we have completely deleted large parts of the subsection or moved them to the Discussion section (under ‘Limitations’), and have now inserted some newly formulated paragraphs. This has hopefully and effectively (in our opinion) much improved the understanding and/or transparency of the design, setting, size, participants and methods of the study – and avoided redundancy.*

**Results**

“I suggest to revise the description of the characteristics of the sample included in the analyses. Please report numbers of individuals at each stage of the study, e.g. numbers potentially eligible, examined for eligibility, confirmed eligible and included in each survey, and numbers included in the combined dataset and numbers analysed,”

*There must be a misunderstanding here. There has not been a multistage recruitment or selection process or analysis procedure. We have simply put together two datasets and study samples, and have analyzed them subsequently as a whole. In one case the entire sample or survey population was used and in the other case a comparable subsample of a nationwide survey population was selected. And by the way, all numbers of individuals can be calculated from the relative frequencies (%) and the size of the (sub)populations (n/N) shown in Table 1. In our opinion it’s not essential or more informative to indicate relative and absolute frequencies. However, we have made some few adaptions and reformulations to hopefully avoid possible misunderstandings.*

“Please also specify sociodemographic data (mean, standard deviation) for the subsamples (278-286). What were the proportions of individuals considered over-indebted and not over-indebted? Explicitly state the share of over-indebted individuals included in the combined dataset (278-280).”

*Do you refer to the characteristics provided in Table 1? In Table 1 for both subsamples relative frequencies (%) of socio-demographic characteristics are already provided. Means and standard deviations make little sense for categorical variables such as sex, nationality, marital status or employment status. And for ordinally scaled variables such as age, educational level or income they are less significant and little informative. And the share of the subsample of over-indebted individuals of the total study sample is noted (“roughly 10%”) and amounts exactly 9.9%. Absolute frequencies (numbers) of the two subsamples are provided in Table 1.*

*To clarify once more: The subsample of 219 individuals comprises completely of over-indebted individuals whereas the reference group of the 1,997 respondents of the Swiss Health Survey are the representatives of the general population who are considered to be largely not overly indebted and therefore taken and treated as the reference group of the non-exposed, not over-indebted. This is now pointed out more clearly in the Methods section (subsection ‘Data and study population’).*

“I recommend to revise the description of findings, specifically with regards to the link between income (income, categorized into no/ low / medium / high income) and health outcomes. The authors mention in the table footer (Table 4) that Odds ratios were “adjusted for sex, age, education, nationality, marital status, employment status” (342). (Why) did the authors not adjust for income? Moreover, the authors describe that “Low or no personal income was associated with a significantly increased probability of and risk for poor SRH, independent of educational attainent or of over-indebtedness or no over-indebtedness” (323-325). They also state “However, there was no a linear and/or significant effect of the amount of income on the other health outcomes examined.” (325-326). In my view, multiple logistic regression analysis is not suitable to examine a linear relationship (or probability) as stated by the authors.”

*You’re totally right regarding the footer. But of course all studied associations were adjusted for income. The whole footnote was simply an error and beyond that incomplete and unnecessary (and therefore only placed at the foot of Table 4 and not below Table 3 likewise). It was wrongly adopted from an old table…*

*Since all covariates or control variables for which the main predictor or risk factor (over-indebtedness) was adjusted were included in the logistic regression analyses and shown in Tables 3 and 4, such a footnote makes no sense at all.*

*And by “linear” we meant a clear gradient or dose-effect relationship. We have reformulated the sentence accordingly.*

*But apart from that, we have chosen to use logistic regression analyses due to the limited level or scale of measurement (ordinal, NOT interval) and/or the inadequate marginal distribution (strongly skewed, NOT normal) of the outcome variables under study. Linear regression analyses in contrast were not suitable since they require interval scaled and normally distributed variables as dependent or outcome variables. We think anyway that logistic regression analyses are absolutely suitable to examine linear or rather dose-effect relationships, particularly when relationships are curve linear, when a gradient is expected and wants to be studied and estimated and/or when predictors (independent variables) are nominal scaled or were treated like categorical variables and, hence, an indicator method was used to contrast the different categories from an indicated reference category as we did.*

“From my point of view, male sex was not significantly associated with poor self-rated health but marked bold in Table 3 (aOR 0.89; CI 0.69-1.14).”

*Yes, you are completely right. This is a mistake and has been corrected now.*

**Reviewer #2:**

“Although this study is on interesting research topic, there is a main methodological concern and limitation which need to be addressed in this present study. This is related to definition of over-indebtedness construct which is dichotomous and lacking of indicators for over-indebtedness. It is important to explain why measuring over-indebtedness indicators were not given priority in designing this present study.”

*Two text passages have been inserted in the Discussion section (under the subsection “Limitations”) to address and discuss this concern as an additional limitation and to justify the chosen study design.*

“Besides that, in the introduction section—the author(s) may highlight why research addressing over-indebtedness is important in Switzerland and why over-indebtedness might be increase in Switzerland?”

*At the end of the Background section (just before the newly formulated research questions), we have now explicitly summarized what we tried to demonstrate all over the introduction, namely why research on this topic and population group is needed and important, particularly for Switzerland.*

“In line no 313--please revise and specify the statement “...main predictor turned out to be the strongest risk or explanatory factor by far…” Please specify IF the author(s) refer to “the poor health outcomes” or something else.”

*Yes, we have referred to Tables 3 and 4 as described and consequently to the four poor health outcomes shown in these tables. But we made this even more explicit now by specifying “of all poor health outcomes studied”.*

“In line no 357-358--please revise and specify the regression analyses authors referring to—do the authors refer to multiple logistic regression?”

*Yes, since multiple logistic regression analyses were the only multivariate statistical methods or regression analyses that we have used or made in this study. We have slightly reformulated the according sentence.*

We are confident now that all required or suggested changes have been made appropriately and according to the reviewers’ comments. We are now looking very much forward to the publication of our manuscript in the PLOS ONE.

Kind regards also on behalf of my co-author,

Oliver Hämmig
