# Supplementary material for: Over-indebtedness and health in Switzerland: A cross-sectional study comparing over-indebted individuals and the general population
Source: PLoS One. 2022 Oct 11;17(10):e0275441. doi: 10.1371/journal.pone.0275441 (PMC9553041; doi:10.1371/journal.pone.0275441)
Supplement: Attachment — Submitted filename: renamed_8a001.docx [file pone.0275441.s004.docx]

Zurich, 5 August 2022

Dear Dr. Useche,

We have now substantially revised our manuscript entitled

**Over-indebtedness and health in Switzerland: a cross-sectional study comparing over-indebted individuals and the general population**

**(PONE-D-21-26584_R1)**

for a second time.

Please find attached the revised manuscript along with our replies to the comments and points raised by the reviewer(s). All corrections, reformulations and additions are highlighted in light blue. The extensive deletions in the introduction and discussion are not marked.

**Reviewer’ comments and authors’ replies**

**Reviewer #3:**

“Important public health paper on over-indebtedness and health in Switzerland.
Paper is lengthy, particularly Introduction and Discussion. Try to short and avoid repetition.”

*We have shortened the Introduction section by about one third (more than one page), and in addition have reduced the text of the Discussion section substantially.*

“Add paragraph on how the covariates were measured (e.g. income) or point to Table 3 and 4 (and add a footnote there for additional description).”

*Done. A paragraph on the control variables was added at the end of the “Measures” section.*

“The Analyses (line 269), first paragraph is stratified by sex for study population and second paragraph for over-indebted. That is unclear, and not only because in the Results the stratification took place for the over-indebted group only. More importantly, why this stratification and why (ultimately) only for the indebted group? Was there an interaction between sex and over-indebtedness?”

*This stratification by sex for the over-indebted individuals only (in Tables 1 and 2) was done simply to better characterize the population of interest and because the two sexes differ greatly as regards their socio-demographic characteristics and their health status – and NOT due to any interaction between sex and over-indebtedness. However, these stratified descriptive analyses for the over-indebted group and not for the comparison group might be a bit confusing. And since the results do not respond basically to any of the research questions and therefore also were not further discussed in the Results or Discussion section we refrain from this stratification and changed the Tables 1 and 2 accordingly.*

“Line 366: you indeed show that the covariates cannot (fully) explain the impact of over-indebtedness. The key thing is to add “fully”, as you have not shown mediation analyses indicating the extent to which the impact of over-indebtedness is explained by the covariates (but not fully, I agree).”

*We agree that some covariates in fact do have an explanatory or predictive effect but at least cannot fully explain the strong negative health impact of over-indebtedness. Therefore we have reformulated the according sentence as suggested.*

“Discussion, line 418-420. Why is the need for a longitudinal study related to the over-indebted people being hard to reach? Does it not fit better with the reversed causation problem (line 400)?”

*We have reformulated and strongly shortened the according paragraph.*

“2nd: Talking about case – control study is confusing, as you seem to imply that the over-indebted are cases and the other are controls. In epidemiological research cases are the one with the disease and controls are the ones without disease. This is confusing (line 420-432).”

*We understand that this might be confusing and have reformulated the whole paragraph and cancelled the conclusion about alternative study designs of a prospective cohort or retrospective case-control study.*

“3rd: paragraph 433-447 switches from a limitation (no variation within over-indebtness), which might or might not be problematic (as there might be differences in “the level of indebtedness”) to a report on a design that you did not use (having only over-indebted group) which is of course good, but one can always imagine more worse designs than your own. The whole paragraph is lengthy and too much words combining pros and cons in confusing way.”

*We have now deleted the whole paragraph.*

**Minor**

Reference for social disparities in Switzerland (line 154).

*The entire paragraph has now been deleted in the course of the text cuts.*

- Methods: tip of the iceberg sentence (line 209): think of having such reflections in the Discussion.

*No / no more (since the Discussion has partly shortened).*

- Methods: reason for over indebtedness (line 211 and further): is that not more Results?
  Type line 250.

*We have now moved the paragraph to the beginning of Results section and have put an introductory sentence in front.*

- Think of having a sentence in Measures saying you have two physical and two mental health outcomes. Otherwise, Table 3 and 4 initially are a bit unclear.

*Done. We have added a sentence on that under the Measures section (just below the description of the over-indebtedness variable).*

- Line 31: better in Measures section on SRH?

*???? (makes no sense; presumably wrong page reference?)*

- Line 315-316: “very poor health status”? You do not know, you only know that they report poor health more often. Formulate otherwise.

*Done.*

- Line 323: “by far”: uncommon in academic writings.

*Done.*

- Line 381: aOR=8.5?

*Correct (and changed accordingly). Thanks very much for detecting this mistake…*

**Reviewer #4:**

“The manuscript significantly improved after this revision. However, introduction and limitation sections unnecessarily long that need to be concise before final decision.”

*Done (see reply to first comment of reviewer 3).*

We thank particularly reviewer 3 for his valuable and detailed comments which we have addressed accordingly and as suggested. We hope that our paper is now finally ready and acceptable for publication in PLOS ONE.

Kind regards also on behalf of my co-author,

Oliver Hämmig
